# Horseshoeing1A paper read before the New York County Veterinary Medical Association.

**Published:** 1895-06

**Authors:** J. H. Ferster


### HORSESHOEING.[1]

BY J. H. FERSTER, V.S.

In consenting to read a paper before this society and its invited guests this evening, I did so, not with the idea of telling you anything new, but rather with the thought that something might be said which would lead to a discussion that would do us all some good. This is the only excuse which I have to make in asking your indulgence, when so many able gentlemen are here to speak to you.

At our last meeting this same subject of horseshoeing was discussed, and some important points were brought forward. Among the most important of these, in my mind, was the point of increasing or decreasing the obliquity of the pastern by elevating the toe. Professor Williams says we increase it, and it has been so taught in our veterinary colleges ever since he made the statement. I believe it to be the most inexcusable error that was ever launched upon any profession in the guise of a theoretical fact, and I am glad that some gentlemen of our day and our city have devoted enough time to the consideration of this point to be able to throw the calcium light of reason upon it, and to take the initiatory step in leading the veterinary profession out of the darkness of this error into a glorious sunlight of truth.

When I was attending college this theory of Professor Williams gave me much trouble. I never could harmonize it with what I thought I found practically to be a fact; and yet, after being repeatedly assured by those in whose opinions I had great confidence that the theory was correct, I gradually came to the conclusion that it must be like some of the ways of Providence—beyond the comprehension of ordinary intellects. In fact, I was repeatedly assured by my classmates, in our discussion of the subject, that it was my intellectual blindness that prevented me from seeing it as Professor Williams saw it. I am glad now to find some of the foremost thinkers of our day sharing in my intellectual blindness.

The obliquity of the pastern is largely regulated by the perforans tendon. If we increase the distance between the points

[1] A paper read before the New York County Veterinary Medical Association.

of attachment of the perforans tendon, without lengthening it, we must increase the tension of that tendon. Now, if that tendon runs in a perpendicular line, we simply increase the tension without altering the direction; but if there be an obliquity of of that tendon the tendency of the increased tension is to straighten it, and if the amount of force brought upon the points of attachment is greater than the resisting force at the point of obliquity the lesser force must yield to the greater, and we have a decreased obliquity. This is what takes place when we elevate the toe.

The importance of understanding whether elevating the toe increases the obliquity of the pasterns or not is demonstrated daily in our practice. We have a horse which stands knuckled, that is, shows an undue perpendicularity of the pastern. To remedy this we must increase its obliquity. How shall we do it? According to the old teachings we should elevate the toe. In practice we do nothing of the kind; but, on the contrary, we elevate the heel, which is, of course, equal to lowering the toe, and we get a tendency to an increased obliquity. Now, is it possible for a man to understand balancing the foot if he does not first understand this point, and understand it not as it has been taught in the past, but understand it as it really is, just the opposite? Here is where reform should begin. If our text-books are wrong, as they surely are, they should be righted, or other text-books substituted. We cannot gather grapes from thorns, or figs from thistles; neither can we teach the coming veterinarians or horseshoers how properly to balance the foot if we begin by giving them an erroneous idea upon this important point. We wish to learn facts that will support us in everyday practice, and I am free to say that I have learned more about preventing and curing lameness by balancing the foot during the past year in which I have been reading Mr. Roberge's book and in conversations with the author, than I learned in the previous ten years of constant study and a course in a veterinary college. The text-book of the future, upon this subject, if it is not Mr. Roberge's work, will be one written upon the lines laid down in his work. When our colleges advance to this idea, then we will largely dispense with our firing-irons, our blisters, and all other forms of counter-irritation, and cure our lame horses in the more humane and more certain manner of balancing the foot. I believe that the next ten years will show greater advance in the science of horseshoeing than has been made in the past fifty years.

Again, it will be very difficult to apply the science of balancing the foot if we do not understand the language of the horse upon this point. A horse pointing from an unbalanced foot always points toward the longest part of that foot, and to stop that pointing we have simply to cut away that part of his foot, or, if this is impossible, to build the opposite part of his shoe enough higher to balance the foot properly.

We are very apt to associate the act of pointing and limping with some abnormal conditions interfering with the usefulness of the animal, but I wish to make the statement here for your consideration that, in my opinion, pointing, and even lameness, are not necessarily an assurance of any abnormal condition of the foot or limb. I have known horses to point while standing, and to limp very perceptibly when moved, in the absence of any abnormal condition at all. Some of you may question this statement and ask for proof. I will cite one case which I find but typical of many. A horse that for nearly a year had been pointing one foot when at rest, and was very lame when moved faster than a walk, that had been pronounced incurable by some of the leading veterinarians of our city (and I do not say this as a reflection upon these veterinarians, as I personally feel like taking off my hat to some of them in acknowledgment of their superior mental development), was shod, experimentally, with a centre-bar shoe, and never took a lame step after that time, except when he so stepped as again to force his foot in the position in which it was before being shod with the centre-bar shoe. Now does it occur to any gentleman present that there was any abnormal condition in this horse while shod in the old way that interfered with his usefulness? If there was, can anyone present explain how it should have assumed a normal condition in a fraction of an hour? I think we all believe that pathological conditions are not regulated so rapidly. No. There was simply an abnormal position and not an abnormal condition, and the moment that abnormal position was righted that moment the horse was right. That horse was simply compelled to favor that limb, because we in our blindness gave him an abnormal bearing upon its articular surfaces. I cite this case for two purposes. First, to bear me out in making the statement that pointing, or even lameness, is not necessarily an assurance of any abnormal condition of the foot or limb, and second, that it may teach you the same lesson it taught me, viz., that balancing the foot is the key to the treatment of lameness, excepting such as may be caused by accidents. I might cite the case of a horse owned by myself, but it teaches the same lessons, with simply a little variation of detail, so I will not take your time with its recitation.

A thorough knowledge of balancing the foot properly is as necessary to the horseshoer and the veterinarian (the opinion of some of our members to the contrary, notwithstanding) as a thorough knowledge of the multiplication table is to a bookkeeper. A bookkeeper who does not understand the multiplication table may keep an accurate record of the daily doings of a firm, but when it comes to balancing his books without a knowledge of the multiplication table he arrives at a correct conclusion only by accident, if he arrives at one at all. And so the horseshoer may be able to make a nice shoe, and nail it to the horse's foot, but without the knowledge of balancing the foot he can benefit the horse only by accident, if he benefit him at all.

The question was asked at our last meeting, "Can a man be an expert horseshoer who is not a practical horseshoer?" If by that question was meant whether a man can understand the scientific part of horseshoing without being able to make with his own hands a shoe, and nail it to the foot, I would answer emphatically, *Yes!* There is a difference between art and science: Art is the ability to make a thing, while science is the knowledge that tells us whether it is properly made or not. The veterinarian who supposes that he must become a practical horseshoer before he can direct the proper shoeing of a horse makes a mistake which reflects very much upon his intelligence, and the practical horseshoer who believes he understands the science of horseshoeing without having thoroughly familiarized himself with the anatomy, etc., of the foot and limb makes an equally dangerous blunder.

How often we hear some horseowner or groom or adviser of some description saying, "Fit the shoe to the foot, and not the foot to the shoe," without having the least idea of the evil such a thing would do if it were actually practised. They say it because they have heard some one else say it. This is one great trouble, also, with too many of our horseshoers and veterinarians of to-day: we are too apt to take some other man's word for anything and not take the trouble to investigate for ourselves. It were better if we would cultivate a spirit of investigation—or doubt, if you choose to call it so. Doubt, and you will think; think, and you will doubt. The veterinary surgeon and the horseshoer should work hand-in-hand, each striving to help the other as they help themselves. Nothing will keep a profession or a trade from improving its status so thoroughly as the failure of its members to investigate new discoveries in its especial line So I say to you all, horseshoers and veterinarians, get out of old ruts and see if things do not move along easier. Investigate before you condemn, and investigate with a desire to get more light, and not with a predetermination to condemn.

But let us return for a moment to that old threadbare saying we referred to a second ago, and which is responsible for a great many lame horses: "Fit the shoe to the foot, and not the foot to the shoe." We see one horse toeing-out and another toeing-in; one horse's heels grow fast and his toes grow slow; another whose heels grow slow and his toes grow fast; one whose inside heel or toe grows faster than his outside heel or toe, etc. Now fit a shoe to all these feet, without fitting the foot to the shoe, and see what kind of a foot you will have in three or six months. If you are a veterinarian and depend upon one of these horses to draw you around in your business, you had better buy a wheel at once or provide some other means of navigation.

I am not one of those who try to lay the blame of all lame horses at the door of the horseshoers, although I believe that a very large majority of lame horses can be cured by proper shoeing, and this large majority are made lame by improper shoeing. But all the improper shoeing is not directly attributable to the horseshoer. How many stablemen or owners bring a horse to the shoeing-shop and direct him to be shod in this or that way, not knowing any reason for so doing, but simply that Mr. Brown or Mr. Smith had one shod that way, and they want the same thing done, and if the shoeingsmith does not do it he loses the work and some other shoeingsmith gets it.

We hear a great deal about the ignorant horseshoer. Well, no one doubts but what there is a great deal of ignorance among horseshoers; but will some gentleman name a calling in which there is not a great deal of ignorance? Look, if you please, at the veterinary profession. Compare it fifty years ago with today, and you will say that there has been a wonderful advance, and yet with all our present learning we are only able to skirt the shore of the vast ocean of knowledge. The wisest among us are those who feel most keenly their need of more enlightenment.

We hear many veterinarians complaining that the profession is not appreciated by the public, but I believe we get, as a profession, all we deserve. Fifty years ago a prominent medical gentleman would have taken it almost as an insult to have been invited to participate with veterinarians in a discussion relative to the public health. To-day leading veterinarians are given prominent positions in such discussions. To-day our own government has recognized our profession by the establishment of a bureau that has accomplished an almost inestimable amount of good for stock-raisers and meat-consumers, and at its head has placed one of our own profession, selected for professional ability and not political preference, of whom every veterinarian (except, possibly, a few soreheads), feels justly proud—Dr. D. E. Salmon—and is constantly showing its interest in our profession by the passage of laws to better its condition. The profession has steadily advanced until there is but a shadow between it and its sister medical profession. The borderland of one reaches within the borderland of the other. Do you ask for more recognition than this? It will be more to our credit if we spend our time in untiring efforts to rise mentally to the occasions constantly being offered to us instead of lamenting that we are not appreciated. How has this change been brought about? Simply by the members of our profession seeking a higher education.

The leaven that has been working in the veterinary profession for the past twenty years is now working among the horseshoers, and will result in the elevation and education of the members of that calling. I have unbounded faith in the ability of the horseshoers to rise to the elevated position awaiting them, and I have this faith because I believe the shoeingsmith of to-day is a searcher after truth; but I believe that he has the prejudices and the teachings of his forefathers to dispel from his mind before he can make much progress in his art.

This leads us to a moment's consideration of the Shoeingsmiths' Bill, which is now, I believe, in Albany. There is only one encouraging feature about the bill, and that is that it demonstrates that horseshoers are awake to the fact that there is a need of improvement in their line. The bill itself is entirely premature and unworthy the consideration of the horseshoers or the veterinarians. The time for education is at hand, but not the time for legislation. As soon as the horseshoers improve their opportunity of becoming thoroughly learned in the anatomy and physiology of the foot and limb then legislation may be proper and will come easy. This bill can only be characterized as one to benefit the few at the expense of the many. I believe there is a greater future in store for the studious, painstaking horseshoer than for any other class of workmen on the globe. It will not be long before ninety per cent. of lame horses will be treated in the shoeingshop instead of in the veterinary hospital, and the shoeingsmith who does not know how to do it, or the veterinarian who does not know how to direct it, will find himself a back number, and, like the back number of many a magazine, valuable only as a curiosity.

Already I fancy I see in the eastern horizon the morning light of a new era in horseshoeing—an era as far ahead of the old as the lightning express is ahead of a jackass for carrying mail through the country. When I see, as I have seen during our last session at college, horses come in so lame that they could scarcely extend one foot in advance of the other, shod in the old way, and see them then and there shod in the new way, and go out about their daily work with no further treatment, then I feel like extending my hand to every horseshoer, to every veterinarian, and to every one who loves the horse, and solicit their assistance to "ring out the old, ring in the new."

---

Between one hundred and one hundred and fifty graduates in Ohio declined to present themselves and their credentials before the Ohio State Board of Veterinary Medical Examiners, and have jointly employed a lawyer to test the constitutionality of the act creating said Board.

